# The SPHN Schema Forge – transform healthcare semantics from human-readable to machine-readable by leveraging semantic web technologies

**DOI:** 10.1186/s13326-025-00330-9

**Published:** 2025-05-08

**Authors:** Vasundra Touré, Deepak Unni, Philip Krauss, Abdelhamid Abdelwahed, Jascha Buchhorn, Leon Hinderling, Thomas R. Geiger, Sabine Österle

**Affiliations:** 1 Personalized Health Informatics, SIB Swiss Institute of Bioinformatics, Basel, 4051 Switzerland; 2 Accenture, Basel, 4051 Switzerland; 3 Swiss Academy of Medical Sciences, Bern, 3001 Switzerland; 4 Independent researcher, Basel, Switzerland

**Keywords:** Healthcare, Semantics, Semantic Web, Schema, RDF, Spreadsheet, FAIR, Web Service, Conversion

## Abstract

**Background:**

The Swiss Personalized Health Network (SPHN) adopted the Resource Description Framework (RDF), a core component of the Semantic Web technology stack, for the formal encoding and exchange of healthcare data in a medical knowledge graph. The SPHN RDF Schema defines the semantics on how data elements should be represented. While RDF is proven to be machine readable and interpretable, it can be challenging for individuals without specialized background to read and understand the knowledge represented in RDF. For this reason, the semantics described in the SPHN RDF Schema are primarily defined in a user-accessible tabular format, the SPHN Dataset, before being translated into its RDF representation. However, this translation process was previously manual, time-consuming and labor-intensive.

**Result:**

To automate and streamline the translation from tabular to RDF representation, the SPHN Schema Forge web service was developed. With a few clicks, this tool automatically converts an SPHN-compliant Dataset spreadsheet into an RDF schema. Additionally, it generates SHACL rules for data validation, an HTML visualization of the schema and SPARQL queries for basic data analysis.

**Conclusion:**

The SPHN Schema Forge significantly reduces the manual effort and time required for schema generation, enabling researchers to focus on more meaningful tasks such as data interpretation and analysis within the SPHN framework.

**Supplementary Information:**

The online version contains supplementary material available at 10.1186/s13326-025-00330-9.

## Introduction

Healthcare data in Switzerland obtained from multiple source systems, with varying standards, quality assessments, and languages, pose significant challenges to data harmonization and integration in research. Achieving interoperability in this context requires a structured and semantically rich framework that can bridge these differences while remaining accessible to diverse stakeholders, including clinicians, data managers, and researchers.

To address these challenges, the Swiss Personalized Health Network (SPHN [1]) has developed the SPHN Semantic Interoperability Framework guided by a three-pillar strategy described in [2] and aligned with the FAIR (Findable, Accessible, Interoperable and Reusable, [3]) principles. The framework is centered around the SPHN Dataset, a structured spreadsheet that defines healthcare semantics in alignment with international standards. This Dataset is complemented by Semantic Web technologies [4, 5], including the Resource Description Framework (RDF [6]) for semantic modeling and data exchange, Shapes Constraint Language (SHACL [7]) constraints for data validation, and SPARQL Protocol and RDF Query Language (SPARQL [8]) queries for data exploration.

While these components form a powerful foundation, their maintenance and extension have proven to be labor-intensive and technically demanding as the complexity of the semantics grows over time, particularly as they are created manually (through Microsoft Excel [9], Protégé [10] or WebProtégé [11]) and require familiarity with Semantic Web technologies.

A range of tools and frameworks have been proposed to streamline semantic modeling. For instance, the Reasonable Ontology Templates (OTTR, [12]) allow users to define reusable ontology design patterns for generating RDF structures; the Linked Data Modeling Language (LinkML [13]) provides a schema language for describing data models to be serialized in different formats such as RDF; and ROBOT [14] supports ontology workflows and ontology-based data integration as a command-line based tool. However, these tools either assume advanced technical knowledge, introduce additional complexity, or do not align with SPHN’s spreadsheet-based process and user base; and therefore, fall short on addressing the specific needs of SPHN.

To solve this problem, a tool that automates the transformation of spreadsheet-based semantics into machine-readable artifacts, while remaining intuitive for users without a background in Semantic Web technologies would be ideal.

We present the SPHN Schema Forge, a scalable and user-friendly web service that automatically formalizes the SPHN Dataset into RDF-based artifacts. This tool supports both data providers and researchers by reducing the technical burden of semantic formalization, ensuring consistency across artifacts and improving overall maintainability. In this paper, we provide background information on the required structure and content of the SPHN Dataset and describe the development and architecture of this solution, detailing how statements originating from both the dataset and the schema are interpreted and processed by the tools underlying Schema Forge.

## Background

### Semantic Web technologies in SPHN

Initially, the SPHN Semantic Interoperability Framework defined semantics in the SPHN Dataset spreadsheet (.xlsx), aligning with international standards and models [15–18]. The tabular format offers an efficient structure for simple datasets and is understood by diverse experts (e.g. clinicians, data analysts, data engineers) given its wide use across scientific domains for data sharing [19, 20]. However, tables struggle to represent complex relationships and hierarchies and are not easily machine processable. This poses a barrier to both interoperability and automated data reuse making them insufficient to support the goals of SPHN.

To address these limitations, Semantic Web standards were adopted, transforming tabular data into more expressive, machine-readable formats, and enabling integration with other standard terminologies and vocabularies [21].

The Semantic Web is an extension of the World Wide Web to enable machine-readable representation and sharing of data and its semantics across systems. At the core of the Semantic Web are a set of standards and technologies developed by the World Wide Web Consortium (W3C) that provide a formal and structured way of representing knowledge [4]. These standards serve as the foundation for enabling interoperability between systems [22–26].

A fundamental building block of the Semantic Web is RDF [6], a graph-based data model designed to represent knowledge as triples. Each triple consists of the subject, a predicate, and an object which results in a directed graph structure that models information about entities and relationships between entities.

RDF Schema (RDFS, [27]) builds on RDF for describing schemas by defining classes, properties, hierarchical structures and relationships between resources. However, RDFS has limited expressivity when it comes to defining more complex relationships and constraints, which is typical when considering biological, biomedical and clinical knowledge. To address these limitations, the Web Ontology Language (OWL, [28]) was introduced. OWL offers a rich language for defining the meaning of concepts and their relationships, including cardinality and value set restrictions, all of which are useful in the context of SPHN.

Although RDF, RDFS and OWL provide the mechanism for representing knowledge as a schema, they do not ensure that the RDF data conforms to specific constraints or expected structures. This is where the Shapes Constraint Language (SHACL, [7]) comes into play. SHACL provides a way to validate RDF data against a defined set of conditions (i.e. shapes), enabling quality control and conformance of the data.

Another crucial component is the SPARQL Protocol and RDF Query Language (SPARQL [8]), a query language designed for retrieving and manipulating RDF data. It allows users to express complex queries and to query multiple RDF data sources at once.

In SPHN, these technologies formalize the SPHN Dataset into the SPHN RDF Schema, ensuring that the semantics are both machine-readable and compliant with the FAIR principles [5]. Over time, the framework evolved to incorporate additional components like SHACL constraints for facilitating data validation, SPARQL queries for data exploration, and even a Hypertext Markup Language (HTML) documentation for a human-readable version of the semantics. These elements collectively form the SPHN Semantic Interoperability Framework, a package that enables researchers to request and analyze standardized data while allowing data providers to validate and structure their data consistently.

### SPHN Dataset

At the heart of this framework lies the SPHN Dataset. All elements of the SPHN RDF Schema are either explicitly defined in the Dataset or derived through well-established conventions and modeling rules, enabling a fully automated transformation. The SPHN Dataset is the main input to the SPHN Schema Forge. It is a structured spreadsheet that provides a detailed description of the semantics defined within the SPHN Semantic Interoperability Framework. It serves as both a reference document for researchers, data providers, clinicians, and other stakeholders, and as primary resource for generating the SPHN RDF Schema.

The SPHN Dataset is developed and maintained by the SPHN Data Coordination Center (SPHN DCC), ensuring that the core semantics are aligned with international standards, consistently versioned, and documented. However, the framework also supports flexibility by allowing individual projects to extend the Dataset to meet their needs.

For validating the framework in real-world settings, SPHN established four large multicentric consortia in key disease areas (Infectious Diseases, Oncology, Pediatrics, and Quality Care), serving as national data streams [29]. These projects can add new concepts, define additional coding systems, or refine value sets to capture study-specific semantics. While this adaptability supports a broad range of use cases, it also introduces complexity for ensuring consistency, traceability, and interoperability across versions and use cases. Hence, strict conventions and guidelines for semantic modeling are defined. The organization of the SPHN Dataset ensures clarity and uniformity, which is crucial for the next step in the process: automatically formalize the Dataset into semantic artifacts.

The SPHN Dataset consists of six distinct tabs:

- **Guideline**: Introduces the Dataset and describes its content including a list of abbreviations, descriptions of the tabs and their respective columns
- **License**: Outlines the licenses applicable to the Dataset and the main resources used
- **Release Notes**: Documents the changes across releases, including a detailed list of newly introduced concepts
- **Metadata**: The metadata for the RDF schema (e.g. prefix, title, description, license)
- **Coding System and Version**: Lists coding systems or standards (e.g. classifications, ontologies) referenced in the Dataset, along with metadata to facilitate their import in the RDF schema and enable the linking to standard codes
- **Concepts**: Lists all concepts and their attributes defined in the Dataset.

Among these, three tabs, ‘Metadata’, ‘Coding System and Version’, and ‘Concepts’ are processed by the SPHN Schema Forge, further described below.

#### Metadata Tab

Metadata is a critical component of the FAIR principles which ensures clarity, provenance and proper documentation of data. In the Dataset, the ‘Metadata’ tab serves to annotate the metadata for the RDF schema. It must be properly filled for both the SPHN and project-specific RDF schemas to be properly interpreted during the RDF schema generation. In SPHN, the following metadata elements are required:

- **Prefix**: The namespace prefix for the RDF schema (e.g. sphn)
- **Title**: A short title of the RDF schema (e.g. The SPHN RDF Schema)
- **Description**: A short description of the RDF schema
- **Version**: The version of the current RDF schema (e.g. 2025.1)
- **Prior version**: Any previous version from which the current RDF schema is derived (e.g. 2024.2)
- **Copyright and license**: Information on intellectual property and reuse possibilities of the RDF schema
- **Canonical and versioned IRIs**: The unique and versioned identifiers of the RDF schema.

#### Coding System and Version Tab

To enhance interoperability, SPHN semantics often reference known external standards (e.g. Anatomical Therapeutic Chemical classification (ATC [15]), Systematized Nomenclature of Medicine Clinical Terms (SNOMED CT [17, 30]), International Statistical Classification of Diseases and Related Health Problems 10th Revision German Modification (ICD-10-GM [18])). This tab records which coding systems are used along with the necessary metadata for linking them in RDF. In SPHN, these standards are used to annotate values in particular contexts or to establish a ‘meaning binding’ to the concepts (i.e. the concept’s definition aligns with a specific code). When a meaning binding or value set is defined with a specific terminology in the ‘standard’ columns of the ‘Concept’ tab, the corresponding terminology must be referenced in the ‘Coding System and Version’ tab: a new line should be created with the relevant metadata.

In the 2025.1 version of the SPHN Dataset [31], a total of 38 coding systems are referenced. To fully leverage the linked data principles of the Semantic Web, it is ideal for these terminologies to be provided in RDF format. However, only 18 were either already accessible in RDF from the provider (e.g. Ontology for Biomedical Investigations [32], Evidence and Conclusion Ontology [33], Orphanet Rare Disease Ontology [34]) or transformed into RDF format by the DCC Terminology Service pipeline [21] and then made available. The metadata for these RDF-available standards are processed in the SPHN Schema Forge to ensure that the RDF schema properly links to these external coding systems. More information on how to fill this tab can be found in the SPHN Documentation [35].

If a project requires a standard not listed, they can extend the list by also specifying in which context it is used. However, it is the responsibility of the project to ensure compliance with any licensing requirements and, if necessary, to develop adequate pipelines for transforming the standard into RDF if not already available.

#### Concepts tab

**Fig. 1:**
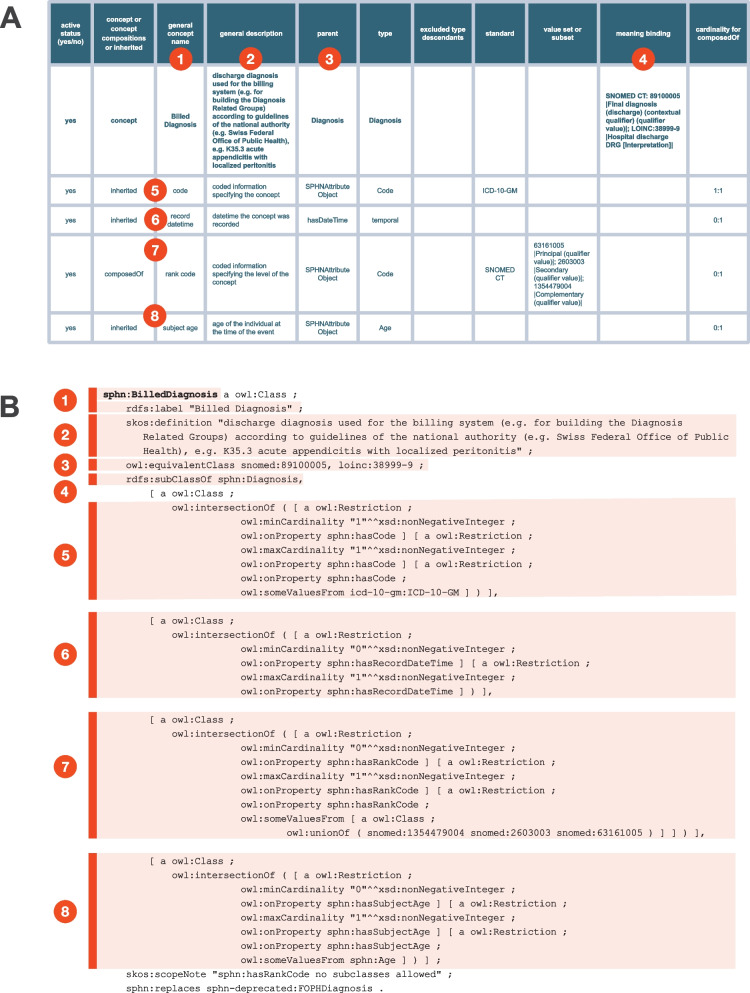
Illustration of the transformation of the 'Billed Diagnosis' concept into its RDF representation. Section A depicts the 'Billed Diagnosis' concept as defined in the SPHN Dataset, while Section B presents its corresponding RDF Turtle representation. Numerical labels in Section A correspond to those in Section B, indicating how specific elements from Section A are interpreted and transformed into their RDF representation

The ‘Concepts’ tab is central for defining semantics. It consists of a single tab with 23 columns (described in Table 1), which must be accurately filled with the list of relevant concepts to enable the automated transformation of semantics into RDF. Figure 1A presents one example of such concept and its properties with the 'Billed Diagnosis' concept as seen in the SPHN Dataset. Each row represents either a concept (class) or a composedOf (attribute), with each column providing specific information about that concept or attribute. In the case of the 'Billed Diagnosis', we are building a concept to represent data about diagnosis at the time of discharge, used for the billing system at a hospital. The ‘parent’ column indicates that this concept inherits from a more generic concept called ‘Diagnosis’. As a result, it also includes all attributes of ‘Diagnosis’ (represented by the rows where the column “concept or concept compositions or inherited” contains the value ‘inherited’). This follows the inheritance rules applied in SPHN where a ‘child’ concept always inherits all attributes from its ‘parent’ concept.

**Table 1:** Overview of columns defined in the ‘Concepts’ tab from the SPHN Dataset and their corresponding RDF interpretation by the SPHN Schema Forge

Column Name	Description	Applies To	Interpretation
release	Defines the release version of the concept (or property)	concept, composedOf	Not translated to RDF. Only used for documentation
IRI (Internationalized Resource Identifier)	Defines a unique resource identifier that is used to refer to the concept (or composedOf)	concept, composedOf	Not translated to RDF. Only used for documentation
active status (yes/no)	Indicates whether the concept (or composedOf) is currently active or deprecated	concept, composedOf	Parsed to identify which rows are active, i.e. marked as ‘yes’
concept reference	Defines the name of the concept to which a composedOf belongs to	composedOf	Parsed to identify which concept the composedOf belongs to
concept or concept compositions or inherited	Specifies whether the entry is a concept (or a composedOf)	concept, composedOf	Parsed to identify whether the row is defining a concept, a composedOf or an inherited composedOf. Rows marked ‘concept’ are represented as an owl:Class while rows marked ‘composedOf’ or ‘inherited’ are represented as properties (either owl:ObjectProperty or owl:DatatypeProperty)
general concept name	The name of the concept (or composedOf)	concept, composedOf	For Concept, the name is parsed to define the concept name where the name itself is in PascalCase representation
general description	The description of the concept (or composedOf)	concept, composedOf	Parsed and represented via the skos:definition predicate of the concept (or composedOf)
contextualized concept name	The specific name of the concept (or composedOf)	concept, composedOf	Not translated to RDF. Only used for documentation
contextualized description	The specific description of the concept (or composedOf)	concept, composedOf	Not translated to RDF. Only used for documentation
parent	The specific parent of the concept (or composedOf)	concept, composedOf	For Concept, the parent name is parsed to indicate the parent, represented via rdfs:subClassOf predicate
type	Specifies the type of the concept (or composedOf)	concept, composedOf	For Concept, the type indicates the semantic type. This is typically the concept name and thus is not translated
excluded type descendants	Lists concept types that should be excluded	composedOf	When the value type for a composedOf is broader, the concept names defined in this row are parsed as a note, via skos:scopeNote predicate, to indicate what specific descendants of the type class are excluded
standard	Indicates the standard from which values can be used for this composedOf	composedOf	When the value type for a composedOf is a Code, the standard defines the coding system from which codes are expected as values. The standard is represented as an owl:someValuesFrom restriction
value set or subset	Defines the specific values that a composedOf can have	composedOf	Defines the list of values, either from a well defined coding system or defined as a custom value set. The values are represented as an owl:someValuesFrom restriction
meaning binding	Defines the meaning of the concept in relation to a code from an external terminology	concept	The meaning binding is expressed via owl:equivalentClass predicate
additional information	Any extra details related to the concept	concept, composedOf	Not translated to RDF. Only used for documentation
cardinality for composedOf	Specifies cardinality of the composedOf	composedOf	The cardinalities are parsed and expressed as an owl:minCardinality and owl:maxCardinality restriction
cardinality for concept to Administrative Case	Specifies cardinality of the concept to the ‘Administrative Case’ concept	concept	The cardinalities are parsed and expressed as an owl:minCardinality and owl:maxCardinality restriction
cardinality for concept to Subject Pseudo Identifier	Specifies cardinality of the concept to the ‘Subject Pseudo Identifier’ concept	concept	The cardinalities are parsed and expressed as an owl:minCardinality and owl:maxCardinality restriction
cardinality for concept to Source System	Specifies cardinality of the concept to the ‘Source System’ concept	concept	The cardinalities are parsed and expressed as an owl:minCardinality and owl:maxCardinality restriction
sensitive (yes/no)	Indicates whether the data represented by the composedOf is sensitive	composedOf	The column is parsed as a boolean and expressed via sphn:subjectToDeIdentification predicate

Each column in the SPHN Dataset defines specific semantic elements, which are mapped to RDF structures such as classes, properties, and constraints. This mapping ensures semantic consistency and facilitates interoperability within the SPHN Semantic Interoperability Framework when the dataset is transformed into an RDF Schema using the SPHN Schema Forge

The type of the ‘Billed Diagnosis’ is captured by the attribute ‘code’ where the coding system used should be ICD-10-GM, the time of diagnosis is captured by the attribute ‘record datetime’, the code that specifies the rank of the billed diagnosis is captured by the attribute ‘rank code’ where the code should be from a fixed set of values from SNOMED CT, and the age of the patient at the time of diagnosis is captured by the attribute ‘age’, which refers to another concept called ‘Age’.

The ‘cardinality of composedOf’ column specifies which attributes are required and, in this case, the ‘record datetime’ is the only mandatory one (i.e. with a cardinality of 1:1).

The ‘value set or subset’ column aims to further refine the valid codes by defining specific subsets of codes allowed for use in a given context. Different scenarios are supported for specifying value sets. When only the ‘standard’ column is filled with the name of a terminology, all codes from that terminology are considered valid. A subset of a terminology can be defined using the keyword ‘descendant of’ followed by a code which indicates that all its child codes are valid. If ‘descendant of’ is not specified, only the explicitly enumerated codes are permitted and their child codes are not considered as valid options. Finally, concepts can hold a ‘meaning binding’ to specific codes from given terminologies to reflect that their meaning aligns with the associated code.

All the rules and conventions are thoroughly described in the SPHN Documentation for the SPHN DCC and projects to consult when building their Dataset [35]. Once the Dataset holds the necessary semantics, it can be parsed by the SPHN Schema Forge to build all the necessary semantic artifacts.

### Methodology

The development of the SPHN Schema Forge follows an iterative and user-centric approach inspired by Agile principles, with a strong emphasis on improving user satisfaction. The approach focused on addressing immediate challenges as they emerged and gradually integrating the solutions into the broader SPHN framework.

The SPHN initiative adopted RDF as a standard for data exchange in its three pillars strategy [2]. While powerful RDF tools were available and effective for technical experts, early feedback from stakeholders highlighted the need for more user-friendly solutions and support beyond the RDF schema alone.

Four main needs raised by the stakeholders included:

1. A simplified modeling process of project-specific concepts and attributes as an extension of the SPHN RDF Schema
2. Defined validation constraints to indicate data modelling errors
3. A set of basic and ready-to-use statistical queries for exploring SPHN and project-extended RDF datasets
4. A user-friendly visualization of both, the SPHN and project-extended RDF schema

These requests were received over an extended period, leading to the development of individual standalone solutions. Later in the process, the solutions for statistical queries and user-friendly visualization were merged following a major redesign, as both relied on the same internal model derived from the RDF schema. For each solution, alternatives have been considered, and research of available tools and frameworks was conducted.

Even with the tools available, generating new releases of the SPHN RDF Schema or project extensions remained cumbersome, as all three tools required to be installed and updated individually. To answer the user’s feedback calling for a more streamlined process, the tools were wrapped together into a simple web service resulting in the SPHN Schema Forge.

The SPHN Schema Forge is built upon a set of guiding principles (and assumptions) that ensure effective implementation and usage. These principles provide a baseline for structuring and transforming semantics in a standardized and interoperable manner.

- **Structured representation**: SPHN Schema Forge relies on a well-structured .xlsx file as a starting point, i.e. the SPHN Dataset. This serves as the source of truth for describing all the necessary concepts, attributes and their semantics. It must adhere to a predefined structure to ensure consistency and usability.
- **Transformation into RDF schema**: The semantics defined in the Dataset are systematically parsed and represented as an RDF schema using W3C standards (e.g. RDFS, SKOS [36] and OWL), enabling machine-readable representation of the semantics while preserving the intended meaning of concepts and attributes from the Dataset.
- **Derivation of SHACL shapes from RDF schema**: The RDF schema must capture all the semantics (and hints) required to derive SHACL shapes, via the SPHN SHACLer.
- **Derivation of Documentation from RDF schema**: The RDF schema should also contain all the necessary semantics (and hints) to derive a self-describing HTML documentation, via the SPHN Schema Doc.
- **Extensibility**: SPHN Schema Forge should facilitate extensibility where SPHN-defined concepts can be extended by users with specific use cases.

**Fig. 2:**
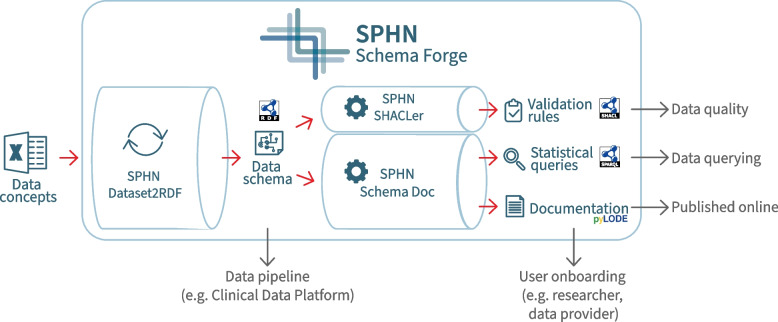
The SPHN Schema Forge pipeline. SPHN Schema Forge integrates the SPHN Dataset2RDF, SPHN SHACLer and the SPHN Schema Doc. This pipeline transforms from an SPHN-compliant Dataset file into multiple outputs by running several tools, including the SPHN Dataset2RDF, SPHN SHACLer and the SPHN Schema Doc. The outputs consist of 1) a RDF Schema used in data pipelines (e.g. Clinical Data Platform), for generating SPHN-compliant data; 2) SHACL validation rules (in RDF Turtle format) to ensure data quality; 3) SPARQL queries for facilitating the query of data for basic statistical insights and; 4) an HTML documentation based on pyLODE, which can be made accessible online to facilitate the onboarding of users to SPHN, as well as projects, and its schema specifications

One thing to note is that the conversion from the Dataset to the RDF schema is a one-way process as shown in Fig. 2. It is not possible to fully reconstruct the original Dataset from the RDF representation because certain information may be consolidated or abstracted during the transformation. This irreversible transformation highlights the need for careful management and preservation of the original Dataset for reference and updates.

### Implementation

The SPHN Schema Forge is a freely available web service accessible at [37]. Its source code, deployed via a CI/CD pipeline, is available on GitLab [38] and is provided under the GNU General Public License v3.0 (GPLv3) open-source license. To use the service, users need to provide an .xslx file that complies with the SPHN requirements and click ‘Run’. Upon successful completion, a set of outputs is generated, consisting of an RDF schema file (in RDF Turtle format), a SHACL file (in RDF Turtle format), a set of SPARQL queries (one query per file) and an HTML file.

The SPHN Schema Forge integrates three main tools for generating the various outputs: SPHN Dataset2RDF, SPHN SHACLer, and SPHN Schema Doc (see Fig. 2). The SPHN Dataset2RDF processes the tabular Dataset, while the SPHN SHACLer and SPHN Schema Doc parses the RDF schema generated by the Dataset2RDF. Each tool described below is licensed under GPLv3 license.

#### SPHN Dataset2RDF

The SPHN Dataset2RDF is the core tool designed to transform an SPHN or project-specific Dataset (in.xlsx format) into its corresponding RDF schema representation. This conversion process follows the SPHN implementation guidelines and standards, ensuring that the resultant RDF schemas are aligned with the semantic interoperability requirements. Based on Python, the SPHN Dataset2RDF makes use of RDFLib [39] for the generation of a valid RDF schema using predicates from RDF, OWL and SKOS for the representation of semantics.

The transformation is achieved as follows: Dataset2RDF parses information provided in each row in the ‘Concepts’ tab and translates the different columns following specific rules to generate an RDF representation. Illustrated with the ‘Billed Diagnosis’ example in Fig. 1, a concept is represented as an owl:Class, with its label defined by rdfs:label and its definition by skos:definition. The cardinalities and restrictions to specific values from either a coding system or a specific set of qualitative values are all represented as owl:Restriction. The list of all columns in the ‘Concepts’ tab parsed by the Dataset2RDF is provided in Table 1.

Projects can use the SPHN Dataset and extend it further with additional concepts (and attributes) according to their use case. To facilitate this approach, we provide the SPHN Dataset Template [40]—a template version of the SPHN Dataset—that can be used as a starting point for projects to define their own concepts. Dataset2RDF supports the parsing of project-specific Dataset but there are some rules and conventions which users must follow to ensure that their semantics are properly defined and thus translated into RDF. For example, a project must indicate their own concepts and attributes by using a project-specific prefix. For the benefit of our users, the full list of conventions is described in the SPHN Documentation [35]. In addition to conventions, there are also a set of well-defined and documented modeling scenarios that are supported by Dataset2RDF. The scenarios serve to highlight the different modeling capabilities based on the expressivity supported by Dataset2RDF. For example, one of the most common modeling scenarios would be the need for a project to extend an existing concept by adding new attributes or modifying the semantics of an existing attribute.

Given the complexity of the content of the SPHN Dataset and the resulting parsing for generating a consistent RDF schema, many checks are implemented to verify that the input file is built correctly. Otherwise, comprehensive errors are provided to the user to resolve the issue originating from their Dataset (see Additional File 1). From a codebase perspective, there are a suite of unit and integration tests that check different modeling scenarios to ensure that the transformations are consistently applied and there are no regressions. The SPHN Dataset2RDF is accessible on GitLab [41].

#### SPHN SHACLer

To facilitate data validation, the SPHN DCC has developed a way to automatically build quality checks based on SHACL which is a language developed for validating RDF graphs. The SPHN SHACLer, made available on GitLab [42], is a Python script that automatically generates such SHACL shapes from a given RDF schema. It can be seen as an interpreter of the RDF schema within a restricted setting: while ontologies typically adhere to an open world assumption, the SHACLer operates under a closed world assumption. This is especially true for patient data, where each patient entry is considered complete when undergoing validation with the SHACL shapes. The validation is using the entailment of RDFS, which automatically traverses downward hierarchical relations, ensuring that class inheritance is covered.

Several types of SHACL constraints, listed in Table 2, covering different aspects of the data, have been implemented in SPHN. For instance, the cardinality restrictions set in the RDF schema (with owl:minCardinality and owl:maxCardinality) are checked via the constraints sh:minCount and sh:maxCount, respectively**. **

**Table 2:** Comprehensive list of constraints used in the SHACLer

Type of constraint	SHACL component used	Verification made by the SHACL
Cardinality constraint	sh:minCount; sh:maxCount; sh:path	Data must comply with the cardinalities defined in the schema (e.g. minimum cardinality is 1)
Class restriction	sh:class, sh:or	Data must comply with the class restrictions applied to a property
SPARQL target constraint	sh:SPARQLTarget	Used when parent and children’s classes have different validation rules (i.e. restrictions applied)
Sequence path	sh:path	Data must comply with restrictions applied on specific paths of a class
Literal type constraint	sh:datatype	Data of data properties data properties must comply with the expected types (e.g. a'hasName'property must be a xsd:string)
Restriction on individuals and instances	sh:in	Existing instance data must be reused (e.g. SPHN value sets are defined as instances and used in specific contexts as values)
Restriction on date times	sh:SPARQLConstraint	A start date time must occur before an end date time in a specific class instance context
Naming convention constraint	sh:SPARQLConstraint	Data should comply with naming conventions defined in SPHN or warnings will be retrieved (using SPARQL constraints)
Validity of terminology codes	sh:SPARQLConstraint; sh:SPARQLTarget	For ATC, CHOP and ICD-10-GM terminologies, SPHN has applied a versioning strategy. Here, it retrieves either info or error messages depending on the validity of a specific version of a code

The table shows for each type of constraints, the SHACL components and targets used and provides information on the type of verification that is made with the generated rule. Note that 'CHOP' refers to the Swiss Classification of Procedures used as a terminology in SPHN and which provided as an RDF file

Generally, the translation of SPHN RDF Schema to a corresponding SHACL shapes is achieved as follows:

1. Extraction of the root classes and schema(s) prefixes
2. Merging the graphs of the SPHN RDF Schema and a possible project-specific RDF schema
3. Producing the metadata for the SHACL file (e.g. creation date, conformance, license, entailment)
4. Loading the object properties and the restrictions for all classes and expanding them through their sub/super class relationships
5. For classes below the root nodes and not having instances in the RDF Schema, creating a sh:NodeShape with:
  - sh:targetClass to the class from the RDF Schema (explicitly only direct instances of the class)
  - sh:closed which is set to false for SPHN only validation and true when validating against both the SPHN and project-specific schema
  - sh:ignoredProperties which includes at least rdf:type, as it is not explicitly listed in the properties section
  - For each property that originates at this class (either through rdfs:domain or as an explicit restriction on the combination of the class and property):
    i. Creating a nested property restriction for this focus class
    ii. Collecting possible minimum and maximum cardinality restrictions on the source/property/target combination and adding them to the nested project restriction.
    iii. Collecting possible target classes or instances from the SPHN RDF Schema. Hierarchical dependencies do not need to be expanded, as the SHACL validation is specified to be executed using RDFS entailment rules. For example, referring to the SNOMED CT root node http://snomed.info/id/138875005 is sufficient to include all classes from SNOMED CT.
    iv. When certain additional hints are given in the schema, such as subclasses are not allowed for certain codes, additional SPARQL constraints are written.
  - For each property chain that originated at this class, creating a nested property chain restriction, as for the normal properties but with incorporating property chains.
6. Each class having instances defined in the schema are value set classes. These value set classes only have instances in the RDF Schema and no further properties originating at this class. For such class a Shape is created, and a reverse instance listing ensures that no value set members are created that are not listed in the schema.

**Fig. 3:**
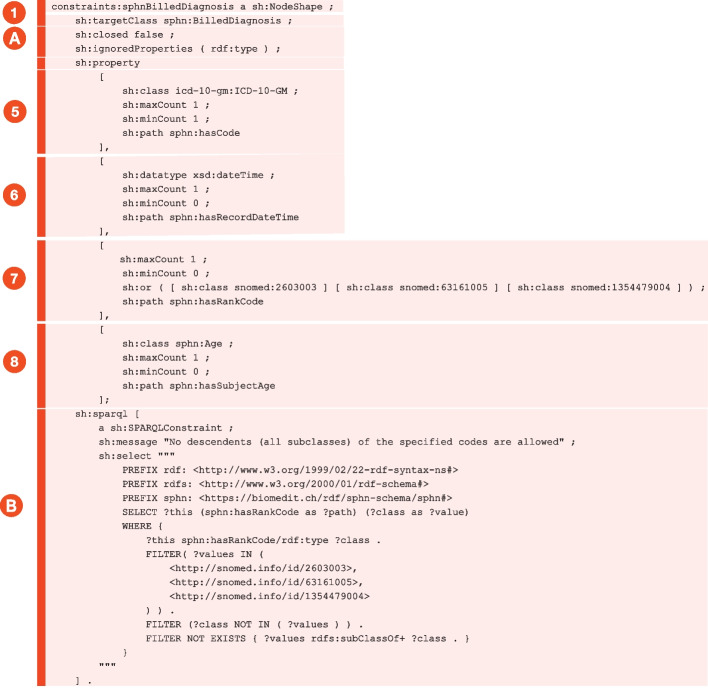
Excerpt of a SHACL shape generated for the 'Billed Diagnosis' concept based on the content of the SPHN RDF Schema. The numbers assigned to specific SHACL shapes in this figure correspond to those previously highlighted in the RDF schema in Fig. 1B

When a data element is not compliant with the schema definitions, the validation with these SHACL shapes would result in an error (sh:Error). Figure 3 provides a correspondence between the RDF schema shown in Fig. 1B and the associated SHACL constraints created:

- A NodeShape is created for each class from the SPHN RDF Schema [5].
- (A) defines the shape being open allowing additional properties to be present. Since the rdf:type is by default used as predicate, it is explicitly listed in the ignored properties. The reason is that the shape will be defined as being closed when project specific extensions are attached.
- (5) enforces the cardinality restriction of sphn:hasCode to be exactly one. In addition, the class is constrained to ICD-10-GM by specifying the ICD-10-GM root node, meaning only instances of this class are valid. Downstream classes don’t need to be included as due to the RDFS entailment.
- (6) specifies the optional sphn:hasRecordDateTime with a dateTime datatype.
- (7) is the direct interpretation of the corresponding part in the SPHN RDF Schema.
- The skos:scopeNote specified at the bottom of the RDF Schema in Fig. 1B, which prevents subclasses of specified codes from being valid, is also parsed by the SHACLer and translated as shown in (B).
- (8) shows a concrete example where sphn:hasSubjectAge must lead to another SPHN class sphn:Age. Here, it is not further checked whether the instance also obeys to the semantics specified for the sphn:Age as there will be a NodeShape for this class.

Additionally, validations are created for certain special properties and under certain conditions. One rule ensures that a hasStartDateTime occurs before or is at least equal to a hasEndDateTime; otherwise, an error is reported. Warnings (sh:Warning) are issued when an instance of a class does not follow the IRI naming convention; or when codes from external terminologies that have undergone a meaning change and are provided in a version where the exact meaning cannot be derived. Finally, codes from external terminologies that have undergone a meaning change and are provided in a version where the exact meaning can be derived, validation rules are generated with a sh:Info severity. This applies for both codes that are still valid in the current release, and codes that are not valid anymore in the current release. We are constantly evaluating whether additional constraints can be derived from the RDF schema to enhance the quality of the data without impeding the raw source of information.

#### SPHN Schema Doc

The SPHN Schema Doc can generate 1) an HTML documentation of the RDF schema for a human readable version of the schema and 2) some statistical SPARQL queries for getting basic information about data conforming to the schema. It is based on pyLODE, a Python-based tool for generating HTML documentation of RDF and OWL ontologies and schemas [43]. The SPHN Schema Doc is accessible on GitLab [44].

Regarding the HTML generation, features have been added to the SPHN Schema Doc to tailor the visual representation to the needs of the SPHN community (e.g. alphabetical ordering of classes and properties, search function panel, table representation of value set restrictions for each class). Additionally, the tool can process images that depict the designed concepts along with direct connections. It integrates the image’s relative paths in the produced HTML at the correct place, so they are rendered correctly in the resulting documentation. These pre-prepared images can be given separately in the SPHN Schema Forge if one would like to render a visualization of their concepts. The HTML for the SPHN RDF Schema is published online at [45].

**Fig. 4:**
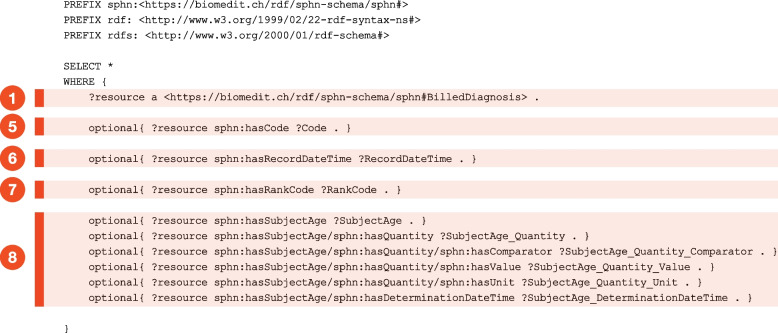
Excerpt of a flattening SPARQL query for the 'Billed Diagnosis' concept. The numbers assigned to specific parts of the SPARQL query in this figure correspond to those previously highlighted in the RDF schema shown in Fig. 1B. For the 'Subject Age' element, the query illustrates how the path is expanded to retrieve nested information about the quantity and the determination datetime

Regarding the generated SPARQLs, they enable users to retrieve relevant statistics by pasting them in any triple store containing the data (e.g. GraphDB [46], Virtuoso [47], Jena Fuseki [48]). These queries were developed in response to researchers’ requests to gain preliminary insights into their data before refining them into more project- and research-specific oriented queries. Technically, the SPHN Schema Doc uses RDFLib to load the RDF schema into a graph and create dictionaries for each Concept, Datatype, and Object Property, including their restrictions. It applies a depth-first search algorithm to traverse the graph from each concept until reaching either a value or a Datatype Property. Based on the retrieved paths, SPARQL queries are constructed for the combination of a source concept and the expanded paths originating at the source. Four kinds of queries are included: flattening (a snippet is shown in Fig. 4 for the ‘Billed Diagnosis’), count of codes, count of instances and ‘min–max’ queries. Flattening queries traverse each concept of the RDF schema to retrieve all connections between a class and its downstream classes, returning all instances linked to the class of interest. If a technical loop occurs (a class referencing itself or reoccurring paths), each loop is traversed only once. The count of codes and the count of instances follow a similar structure but return only aggregated counts of codes (only the property hasCode is parsed in the count of codes) or data instances, respectively. Finally, the ‘min–max’ queries are particularly useful for numerical values or datetimes. They return the minimum and maximum values found in the data for these attributes. All queries released with the SPHN RDF Schema are made available in git, under the quality_assurance folder [49].

## Results

The SPHN Schema Forge [37] is an openly accessible web service that takes an SPHN-compliant Dataset file as input and generates an SPHN-compliant RDF schema (in RDF Turtle format), SHACL shapes (in RDF Turtle format), statistical SPARQL queries and, an HTML documentation within minutes. This provides SPHN projects with a comprehensive package to share with data providers, enabling the request of data that adheres to and valid against the defined and interoperable schema. This ensures flexibility and adaptability for various research needs. Additionally, data users have access to ready-made queries to obtain general statistics about the data they receive. The SPHN Schema Forge implements a suite of end-to-end tests to ensure that the transformation behavior and outputs are consistent across the various components.

It should be noted that SPHN Schema Forge uses and provides content from SNOMED CT. Hence, users of the web service must register for an affiliate license for SNOMED CT, currently free of charge for Swiss users.

Since 2022, for each release of the SPHN RDF Schema and related semantic artifacts, the SPHN DCC has used the SPHN Schema Forge to generate all these files and make them publicly available in the GitLab repository [49]. This automation step has drastically reduced the time spent on building these files, from days to just minutes. The four national data streams have also extended the SPHN Dataset to include their own semantics by using the SPHN Dataset template and the SPHN Schema Forge for generating semantic artifacts. Feedback collected during the implementation and testing phasing led to key improvements such as the adoption of a web-based implementation to avoid the need for a local installation, the integration of SHACL shapes based on data quality validation needs and generally the need for a more intuitive tool for building semantic web content for ‘non-ontologist’ users.

## Discussion

There are several tools and frameworks available for defining data models and schemas (e.g. OTTR, LinkML) that offer useful features and expressive capabilities for defining and maintaining schemas. These approaches are particularly well-suited to contexts where users are familiar with semantic web technologies and comfortable working with machine readable formats (JSON, YAML, XML) and templating mechanisms. However, for many users, these tools come with a steep learning curve.

In the case of SPHN, the primary users are domain experts who primarily engage with data (and its underlying semantics) through tabular formats like spreadsheets. Reducing friction and ensuring broad participation—without requiring prior knowledge of semantic web technologies—was a top priority. At the time, we couldn’t identify a solution that was both feature-rich and user-friendly. As a result, we developed a custom solution tailored specifically to our users and their workflows. This purpose-built approach emphasizes usability and familiarity while maintaining alignment with FAIR principles. As we built and refined this solution, we uncovered several needs unique to our community which in turn has shaped how we approach tool development.

### Automation does not remove the need for human expertise

The SPHN Schema Forge benefits the SPHN community particularly those who are novice in using Semantic Web technologies. While this tool accelerates the technical implementation, human expertise with domain knowledge is still needed to ensure semantic accuracy and feasibility. Making real-world data accessible for research purposes requires a balance between automation and expert-driven semantic definition.

In SPHN, the automation allows to better focus on semantics and enriching the SPHN Dataset. The governance of the SPHN Dataset is a collaborative process that ensures that the Dataset remains up-to-date, accurate and aligned with evolving needs of research and the availability of new data types and modalities. The responsibility for updating and maintaining the Dataset lies with the SPHN DCC, in close collaboration with the Semantics Working Group (WG) that consists of subject matter experts and data engineers from participating data provider institutions, such as university hospitals. These experts design, build, and refine concepts to accurately reflect real-world clinical data. They discuss, evaluate and refine the concept collaboratively. The finalization of concepts and their definitions occurs only when there is agreement among the WG members. This ensures that the Dataset reflects a shared understanding across institutions.

The SPHN DCC provides guidance and oversight throughout this process to maintain alignment with SPHN’s overall goals for semantic interoperability. A new version of the Dataset, incorporating updated concepts and definitions is released annually, following a structured format (e.g. the latest version being 2025.1), along with other derived semantic artifacts. The standard release period is at the beginning of each year, which gives the participating institutions a predictable schedule for integrating the new release.

For projects extending the SPHN Dataset and the derived semantic artifacts, significant effort is required to meet the quality standards of SPHN. They are required to follow established rules and conventions to ensure a correct schema generation. To support this process, the SPHN DCC provides on-site training, regular meetings, documentation (e.g. [35]), instructional videos [50], and even tailored error messages in the tools when transforming semantics (see Additional File 1). Projects are encouraged to align their extensions with existing concepts and reuse attributes whenever possible. When introducing new concepts, projects would typically discuss them within their consortium. The SPHN DCC can offer recommendations on structuring them to fit within the broader semantic framework.

The SPHN initiative fosters collaboration among all stakeholders to prevent semantic fragmentation and promote long-term integration of project-driven extensions into future versions of the SPHN Dataset.

### Validation beyond schema compliance

Ensuring high-quality data typically requires having robust validation tools. Data generated in the context of SPHN must adhere to the definitions of the SPHN RDF Schema. However, even with well-defined specifications, there is room for interpretation during data generation. Errors may only become apparent during data analysis. Additionally, the various systems used within and across hospitals complicate the data standardization process. In some cases, the source data fails even to provide the level of detail expected in SPHN.

The SPHN SHACLer was developed to streamline the validation process for SPHN-related data. By automating the generation of validation rules, the SHACLer provides the means to ensure compliance with the RDF schema. Data compliance makes sure that the data matches the specifications and restrictions of the schema. For instance, the unit associated with the quantity of an oxygen saturation is a percentage.

Beyond schema compliance, data quality can encompass multiple dimensions [51] like correctness (ensuring data represents the right information, e.g. weight and height of a patient), accuracy (ensuring precision of the data, e.g. ‘cancer diagnosis’ is less precise than ‘invasive ductal carcinoma’ diagnosis), and completeness (ensuring all metadata is provided, e.g. full birth date versus year only). These aspects are not covered by the rules generated by the SHACLer as they may vary across different projects.

Projects can customize their SHACLs by adding constraints to ensure the correctness or accuracy of the data, such as flagging inconsistent values or enforcing certain levels of precision. For instance, a pediatric study could define a rule to flag patients over eighteen. While this task requires familiarity with SHACL, such extensions help data providers avoid sending irrelevant data. However, validating data completeness remains challenging as it is inherently dependent on data availability at the source.

## Conclusion

The SPHN Schema Forge web service automates the transformation of health-related semantics, defined in a tab-delimited format, into semantic web standards. By streamlining the process, the tool reduces the manual workload for the SPHN Data Coordination Center and makes it easier for projects to generate FAIR-compliant and exchangeable schemas and data. Beyond SPHN, the SPHN Schema Forge has the potential to contribute to broader efforts in health data interoperability by facilitating the adoption of semantically described machine-readable data representations. However, while the SPHN Schema Forge simplifies and automates the generation of semantic artifacts, data modeling and the definition of the underlying semantics remain a manual, expertise-driven and time-consuming process. Future developments could explore possibilities for automated quality validation and enhanced user interaction in these areas.

Overall, the comprehensive package produced by the tool helps both data creation and consumption processes though well-defined semantics coupled with validation and exploration capabilities. Hence, the SPHN Schema Forge advances the goals of SPHN for harmonizing and utilizing data across the Swiss healthcare landscape, laying a foundation for future initiatives in semantic data management.

### Availability and requirements

Project name: SPHN Schema Forge.

Project home page:

· Web service: https://schemaforge.dcc.sib.swiss/

· Source code: https://git.dcc.sib.swiss/sphn-semantic-framework/sphn-schemaforge

Operating system(s):

· Web service: Platform independent

· Source code: MacOS, Linux, Windows (recommended with WSL2)

Programming language: Python, HTML, CSS.

Other requirements: Registration to SNOMED CT license.

License: GPLv3.

Any restrictions to use by non-academics: None.

## Supplementary Information

Additional file 1. List of possible errors displayed in the SPHN Schema Forge log and their interpretations. Dataset2RDF list of errors provided to the user. To mitigate the risks of having datasets not compliant with the SPHN rules, the SPHN Schema Forge generates a report for the user. This report contains error messages produced by the Dataset2RDF tool. These error messages aim to guide the user in resolving potential issues. Additional File 1 shows the error messages that may appear in this report and their interpretations. Each error specifies the line in the Dataset where it occurred. The term 'concept' refers to the semantic element of interest defined in the Dataset, which is translated as a 'Class' in RDF. Similarly, the term 'composedOf' corresponds to attributes defined in the Dataset, which are translated as 'Properties' in RDF.

## Data Availability

No datasets were generated or analysed during the current study.

## Abbreviations

ATC: Anatomical Therapeutic Classification
DCC: Data Coordination Center
FAIR: Findable Accessible Interoperable Reusable
HTML: HyperText Markup Language
ICD-10-GM: International Statistical Classification of Diseases and Related Health Problems 10th Revision German Modification
LinkML: Linked Data Modeling Language
OWL: Web Ontology Language
OTTR: Reasonable Ontology Templates
RDF: Resource Description Framework
RDFS: Resource Description Framework Schema
SHACL: Shape Constraint Language
SNOMED CT: Systematized Nomenclature of Medicine Clinical Terms
SKOS: Simple Knowledge Organization System
SPARQL: SPARQL Protocol and RDF Query Language
SPHN: Swiss Personalized Health Network
W3 C: World Wide Web
WG: Working Group
WSL2: Windows Subsystem for Linux 2
.xlsx: Excel Spreadsheet in XML
